# Multiple Long‐Term Conditions, Co‐Long‐Term Conditions and Polyvascular Disease: Considerations for Evidence Synthesis and Meta‐Analyses

**DOI:** 10.1002/cesm.70027

**Published:** 2025-04-03

**Authors:** Gillian Mead, Alex Todhunter‐Brown, Ukachukwu Abaraogu, Amanda Barugh, Arohi Chauhan, Juan Erviti Lopez, Valery Feigin, Jaya Singh Kshatri, Atsushi Mizuno, Sanghamitra Pati, Jackie Price, Rui Providência, Gerry Stansby, Rod Taylor, David J. Williams, James M. Wright, Simiao Wu, Leon Flicker

**Affiliations:** ^1^ Usher Institute, University of Edinburgh Edinburgh UK; ^2^ Glasgow Caledonian University 70 Cowcaddens Road Glasgow G4 0BA UK; ^3^ Division of Biological Sciences and Health School of Health and Life Sciences University of the West of Scotland Scotland UK; ^4^ Centre for Translational and Implementation Research & Department of Medical Rehabilitation University of Nigeria Enugu Nigeria; ^5^ University of Edinburgh Edinburgh UK; ^6^ South Asian Institute of Health Promotion Bhubaneswar Odisha India; ^7^ Innovation and Organization Unit, Navarre Health Service Pamplona Spain; ^8^ National Institute for Stroke & Applied Neurosciences, AUT University 55 Wellesley Street East Auckland City New Zealand; ^9^ Indian Council of Medical Research, Regional Medical Research Centre Bhubaneswar India; ^10^ Department of Cardiovascular Medicine St. Luke's International Hospital Tokyo Japan; ^11^ Indian Council of Medical Research New Delhi India; ^12^ Institute of Health Informatics University College London 222 Euston Road London NW1 2DA UK; ^13^ Freeman Hospital, Newcastle Hospitals NHS Foundation Trust Freeman Road, High Heaton Newcastle upon Tyne NE7 7DN UK; ^14^ School of Health and Wellbeing University of Glasgow Clarice Pears Building 90 Byres Road Glasgow G12 8TB UK; ^15^ RCSI University of Medicine and Health Sciences Dublin Ireland UK; ^16^ University of BC Vancouver BC Canada; ^17^ Department of Neurology, West China Hospital Sichuan University Chengdu 610041 China; ^18^ University of Western Australia 35 Stirling Highway, Crawley Perth 6009 Australia

## Abstract

Cochrane's scientific strategy for 2025 to 2030 has four research priorities, including improving the lives of people living with multiple chronic conditions. The purpose of this article written by the Cochrane Thematic Group in Heart, Stroke and Circulation is to explore considerations around multiple chronic conditions (also referred to as ‘multiple long‐term conditions’ i.e. two or more long‐term conditions) in systematic reviews. Rather than using the term ‘comorbidity’, we introduce a new term ‘co‐long‐term conditions’. We also explore how to define ‘polyvascular disease’. We suggest that review authors consider co‐long‐term conditions and multiple long‐term conditions in their reviews e.g. extract data about how primary studies address co‐long‐term conditions, perform subgroup analyses according to presence or not of co‐long‐term conditions, and include a section in the discussion about how well participants with co‐long‐term conditions were represented in the primary studies. This is especially pertinent for reviews addressing heart, circulatory or stroke disease, and polyvascular disease.

## Graphical abstract

Multiple long‐term conditions are becoming more common as the population ages, and many patients with an index condition have additional conditions (which we propose calling ‘co‐long‐term conditions’). This article provides practical advice on methodological considerations for review authors performing reviews in people with multiple long‐term conditions and ‘co‐long‐term conditions’.

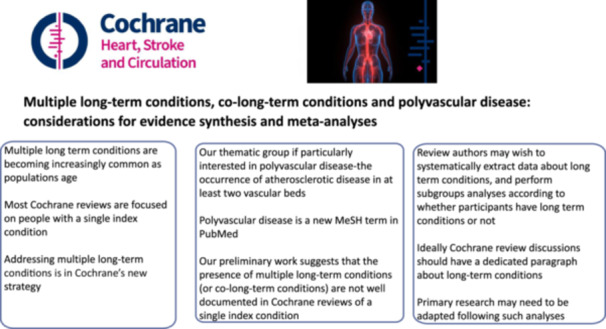

Cochrane's scientific strategy for 2025 to 2030 has four research priorities, including improving the lives of people living with multiple chronic conditions. The purpose of this article is to explore considerations around multiple chronic conditions (also referred to as ‘multiple long‐term conditions’ i.e. two or more long‐term conditions) for systematic reviews. It is well known that as a result of ageing of the world's population, multiple long‐term conditions (MLTCs) are becoming increasingly common [1, 2]. This rate of change is more rapid in low and middle income countries. People with MLTCs have poorer outcomes than those with a single condition [3]. There are several synonyms for MLTCs including multimorbidity, polymorbidity, polypathology, multiple chronic conditions or multiple pathologies. The terms MLTCs and “multimorbidity” (defined as the concurrence of two or more chronic conditions) are in most common use. However, the term “multimorbidity” is not appealing to patients as it implies that they are in poor health, there is little that can be done about their conditions and that they might die soon [4]. For these reasons, the term MLTCs is recommended as this conveys a sense of longevity and hope.

In comparison, the term “comorbidity” refers to a chronic condition that occurs in addition to a chronic index condition [4] e.g. depression in people with stroke. Multimorbidity and comorbidity have had separate MeSH headings since 2018 [5], thereby facilitating literature searches in this area. Despite the shift to using the term MLTCs rather than multimorbidity, the term “comorbidity” is still in use. For consistency, we suggest that the term “co‐long‐term conditions“ should be adopted in the future. We use “co‐long‐term conditions” (CLTCs) within the remainder of this paper, rather than co‐morbidity. If a patient has an index condition, and one additional condition, this additional condition would be the CLTC.

Cochrane was established in 1992 and has pioneered the methodology to perform high quality, rigorous, unbiased analyses of all available evidence to produce “trusted” reviews which are then incorporated into clinical guidelines to improve clinical practice. Cochrane's new strategy for 2025 to 2030 has four scientific priorities; one of which is to improve the lives of people living with MLTCs [6]. There are already several Cochrane reviews of interventions in people with MLTCs as the “index condition” [7, 8, 9], and at least one non‐Cochrane overview of reviews; which identified 30 systematic reviews and 464 unique underlying studies [10]. As the number of randomized controlled trials in people with MLTCs as the index condition increases, further evidence syntheses will be needed, including, for example, umbrella reviews [11]. However, although some reviews include the phrase ‘MLTCs’ in their title, many of the included trials are conducted in people with a single index long‐term condition, with trial inclusion criteria allowing the co‐occurrence of one or more additional long‐term condition [7]. Arguably, true MLTCs reviews should include trials which allow any combination of core index long‐term conditions.

Systematic reviews in people with MLTCs as the index conditions are important, but the majority of Cochrane's 9000 existing reviews are in people with a single index condition. Review authors extract information about inclusion and exclusion criteria of the included studies, and report characteristics of recruited patients. There will therefore an enormous amount of information in Cochrane reviews about CLTCs. However, there is not yet a standard way to report CLTCs or to discuss the generalizability of the evidence. Furthermore, authors generally do not perform subgroup analyses according to the presence or absence of comorbidities.

The Cochrane thematic group in Heart, Stroke and Circulation was established in April 2023 and has a vibrant community of over 1500 members, led by a Board of health care professionals, systematic review experts, and consumers. CLTCs and MLTCs are foci of our planned work. These are particularly relevant for our thematic group as arterial vascular diseases frequently cluster in the same individuals, and significantly contribute to the development and progression of atherosclerotic disease due to sharing the same risk factors e.g. genetic factors, smoking, obesity, hypertension, diabetes, low/inadequate physical activity and sedentary behavior. We are particularly interested in polyvascular disease‐the occurrence of atherosclerotic disease in at least two vascular beds [12, 13], not least because the presence of polyvascular disease is linked with poorer health outcomes and greater costs. However, there is not yet consensus yet about whether atheromatous disease has to be ‘clinically significant, and if so how to define ‘clinically significant’. ‘Clinically significant’ could be defined by the presence of symptoms or not, or by the appearance on vascular imaging (smooth or ulcerated plaque, e.g.), or by the haemodynamic level of a stenosis. A further problem is that major artery stenoses may be asymptomatic, but identifying these asymptomatic conditions is relevant for primary prevention. Asymptomatic disease may be the first presentation of myocardial infarction, sudden cardiac death, or stroke. Although some authors suggest that a 50% stenosis should be used to define “clinically significant” [12, 14], vascular disease is not purely a hydraulic/haemodynamic problem. The atherosclerotic plaque has “a life and biology of its own”, and 60%–70% of myocardial infarctions result from rupture of small to moderate plaques causing less than 50% stenosis before the acute event [15, 16].

Evidence syntheses in polyvascular disease are in their infancy. We are pleased to report that our request to PubMed to include polyvascular disease as a MeSH term (13th November 2024) has been approved. Previously, researchers had to search using a range of different terms including multivessel disease, multisite atherosclerosis, systemic atherosclerosis, and multisite artery disease. We hope that the additional of polyvascular disease as a MeSH term should make it easier for researchers working in this field to identify relevant publications. Developing an appropriate definition of polyvascular disease through Cochrane, as outlined above, and working toward a consensus will be important for moving this field forward, not just for evidence syntheses but for observational studies (e.g. epidemiology, data linkage, diagnostic test accuracy studies), clinical trials and audits. It is likely that some flexibility of definition will be needed‐depending on the purpose e.g. etiological research, diagnostic tests, and interventions aimed at primary or secondary prevention. The key thing is for researchers and clinicians to consider what definition is most appropriate and then state this any primary or secondary research.

What needs to happen next? Cochrane groups could systematically examine their existing reviews to evaluate how CLTCs are currently reported and whether considerations about generalizability take into account the presence (or not) of MLTCs or CLTCs. Also, recommendations for future research should consider an MLTC paradigm and consider how future research should be designed to ensure that people with MLTCs and CLTCs are properly represented.

The Cochrane Thematic Group in Heart Stroke and Circulation intends to investigate a sample of our Cochrane reviews and determine whether the individual included studies involve people with CLTCs, or whether this information is not be ascertained. We also aim to analyze the discussion of these reviews to determine whether the authors addressed the generalizability of the results of the review to people with polyvascular disease and MLTCs and whether they consider the harms of treatment. For example, people with MLTCs are often taking multiple drugs (polypharmacy) and so drug–drug interactions, and drug‐disease interaction are likely to be common. Adverse events (e.g. falls, cognitive impairment) are likely to be more common in people with MLTCs. We also want to report how authors identify gaps in research within the MLTC paradigm.

To inform this study plan, we pre‐specified in a systematic review of exercise interventions for stroke, coronary heart disease and peripheral arterial disease, commissioned by National Institute of Health Research in UK, and performed by NESSIE (NIHR Evidence Synthesis Scotland Initiative) that we would perform prespecified subgroup analyses according to the presence or not of CLTCs [17]. Preliminary results suggest that CLTCs are not well reported in the included primary studies. When published, this review will be a useful blueprint for other systematic reviewers.

We believe further work is required to standardize how Cochrane approaches the reporting of comorbidities in its reviews. This will require considerable consultation regarding definitions, search methods, domains of interventions, and outcome assessment. Methodological research should be done, with patient and public involvement, to develop a formalized structure for extracting information about CLTCs in Cochrane review of a single index condition. Such work would be highly relevant to Cochrane's new scientific strategy.

In the meantime, we suggest four basic points that authors should consider when writing systematic review protocols in a single index condition.

a. Systematically extract data about whether the included studies intended to recruit or exclude people with CLTCs. This will require reviewers to state how they will operationalize CLTCs.
b. Include indicators to the inclusion criteria of primary studies to specify whether data on the pattern of CLTCs are available. These data can be tabulated, and any gaps in reports should be discussed. For some patient groups, where CLTCs are uncommon, this approach might not be relevant, and so review authors could explicitly state that consideration of CLTCs is not relevant.
c. Consider subgroup analyses for people with CLTCs in addition to the index condition. This could apply to both the benefits and harms of treatments. If these analyses are planned but cannot be performed because of the way that data are reported, this should be stated.
d. Include a dedicated paragraph in the discussion section, about whether people with CLTCs were properly represented in the review compared with relevant epidemiological data. Review authors may need to find reliable data from epidemiological studies about the pattern of CLTCs in people with the index condition which they are reviewing.

The importance of CLTCs in the context of single‐vascular disease outcomes remains underrecognized, despite their known prognostic relevance. In particular, numerous evidence syntheses have demonstrated that CLTCs are associated with worse outcomes [18, 19]. Therefore, interpreting evidence synthesis data without considering CLTCs carries a risk of being misleading. However, whilst these are very important considerations for analysis, we are aware that this could add to the workload for Cochrane review authors. Ensuring high quality reviews without creating too burdensome a process is an important consideration for Cochrane as it implements its new strategy which includes MLTCs.

Clinical trialists often rely on systematic reviews—including Cochrane reviews—when designing primary research to test the effectiveness of new treatments. If systematic reviews have addressed the issues of CLTCs, then trialists will be able to better design their research to ensure that it is more generalizable. For trialists considering research in people with MLTCs as the index condition, how MLTCs is defined is a crucial consideration. MLTCs are an umbrella term for highly heterogeneous populations, and the definition depends entirely on the individual conditions they have included when building the construct of MLTC.

We hope that this short paper has alerted review authors to the importance of MLTCs and CLTCs in evidence syntheses, and that our suggestions for addressing these in reviews will help move the field forward. Review authors and other researchers who are interested in methodological research in this area are welcome to contact our thematic group to discuss collaboration in this area.

## Author Contributions

**Gillian Mead:** conceptualization, writing – original draft, writing – review and editing, funding acquisition, project administration. **Alex Todhunter‐Brown:** conceptualization, writing – original draft, writing – review and editing. **Ukachukwu Abaraogu:** conceptualization, writing – review and editing. **Amanda Barugh:** conceptualization, writing – review and editing. **Arohi Chauhan:** conceptualization, writing – review and editing. **Juan Erviti Lopez:** conceptualization, writing – review and editing. **Valery Feigin:** conceptualization, writing – review and editing. **Jaya Singh Kshatri:** conceptualization, writing – review and editing. **Atsushi Mizuno:** conceptualization, writing – review and editing. **Sanghamitra Pati:** conceptualization, writing – review and editing. **Jackie Price:** conceptualization, writing – review and editing. **Rui Providência:** conceptualization, writing – review and editing. **Gerry Stansby:** conceptualization, writing – review and editing. **Rod Taylor:** conceptualization, writing – review and editing. **David J Williams:** conceptualization, writing – review and editing. **James M wright:** conceptualization, writing – review and editing. **Simiao Wu:** conceptualization, writing – review and editing. **Leon Flicker:** conceptualization, funding acquisition, writing – review and editing.

## Conflicts of Interest

The authors declare no conflicts of interests.

## Peer Review

The peer review history for this article is available at https://www.webofscience.com/api/gateway/wos/peer-review/10.1002/cesm.70027.

## Data Availability

The authors have nothing to report.
